# The Double-Edged Sword of Immunosuppressive Therapy in Kidney Transplantation: A Rare Case Report of Pulmonary Mucormycosis Post-Transplant and Literature Review

**DOI:** 10.3389/fmed.2020.00500

**Published:** 2020-09-23

**Authors:** Hengcheng Zhang, Ke Wang, Hao Chen, Li Sun, Zijie Wang, Shuang Fei, Ruoyun Tan, Min Gu

**Affiliations:** ^1^Department of Urology, The First Affiliated Hospital of Nanjing Medical University, Nanjing, China; ^2^Transplantation Research Center, Brigham & Women's Hospital and Harvard Medical School, Boston, MA, United States

**Keywords:** case report, pulmonary, infection, mucor, immunosuppressant, mucormycosis, kidney transplantation

## Abstract

Immunosuppressive therapy is improving the graft survival of kidney transplant recipients and increasing the potential risk of infection. Pulmonary mucormycosis is a rare post-operative infection complication characterized with rapid deterioration and high mortality. In this case, a 33-year-old patient underwent a kidney transplantation with regular immunosuppressive therapy. Soon, 38 days post-transplant, pulmonary patchy shadows can be seen in the radiological examination and rounded into a large cavity formation with splenic rupture 25 days later. The diagnosis of mucormycosis was confirmed by lung biopsy and spleen histopathology. This case is a reminder that early diagnosis is imperative, meanwhile, rational antifungal therapy, timely elimination of immunosuppressants, and alternatively, abandoning the graft should be prudently assessed in the treatment of mucormycosis.

## Introduction

With the use of immunosuppressants, the incidence of rejection after kidney transplantation has dropped considerably in the past few decades (1), which has brought benefits to end-stage renal disease patients and contributes to prolonged graft survival (2, 3). However, as a double-edged sword, the immunosuppressive agents also dramatically reduce the immunity of patients to viruses, bacteria, and other pathogens, thus increasing the risk of peri-operative infection (4, 5). It has been demonstrated that the pulmonary fungal infection is one of the major types of growing infections after renal transplantation, associated with insidious episodes, rapid progression, and severe disease (6, 7). Among these mycoses, aspergillosis is the most common fungal pathogen accounting for more than half of cases (8). However, pulmonary mucosal infections in kidney graft recipients, characterized by rapid progression, high mortality, poor prognosis, and diagnostic difficulties, are rare, occur much less frequently than aspergillosis, and are seldom reported in detail (9, 10).

**Figure 1:**
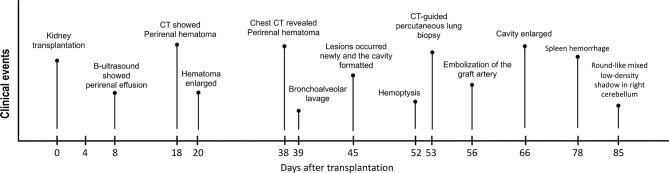
The clinical course of the patient. The pivotal clinical events after surgery of this kidney transplant recipient are showed in this figure.

In the case report, we described a kidney transplantation patient who suffered grievous pulmonary mucormycosis with further deterioration, followed by concurrent splenic infectious foci (Figure 1). We reviewed the relevant references, discussed the course of this case and put forward some reflections on the treatment of peri-operative pulmonary mucor infection in kidney transplantation recipients.

## Case Presentation

A 33-year-old man with end-stage renal disease who underwent 1 year of hemodialysis was admitted to our center for renal replacement therapy. After comprehensive related examinations and the exclusion of contraindications, he received a kidney transplantation from a donor after cardiac death with negative pathogen culture results of blood and kidney lavage fluid. The surgery went smoothly with the cold-ischemia estimated at 12 h along with a warm-ischemia at 10 min. Prevention of infection with linezolid, caspofungin was performed after the operation routinely. We treated this patient with 20 mg basiliximab pre-transplant and then 20 mg basiliximab on the fourth day after transplantation for immunosuppression induction. Considering the long duration of delayed graft function (DGF), we also used the antithymocyte globulin to prevent acute rejection afterward (in total 175 mg, equal to 3.5 mg/kg). The consecutive immunosuppressive therapy included tacrolimus, mycophenolate mofetil, and methylprednisolone.

**Figure 2:**
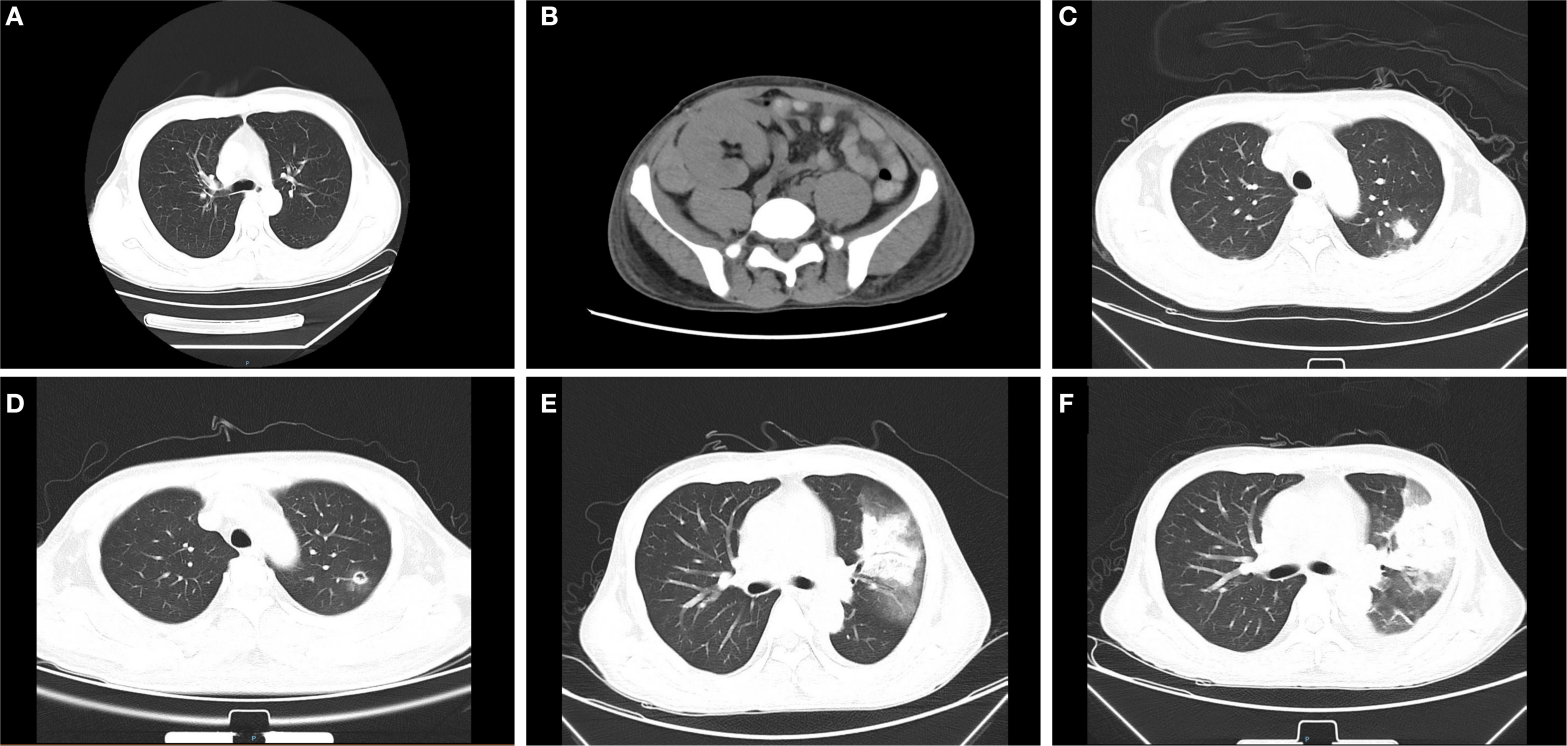
Abdominal and chest computed tomography (CT) scans before and after mucor infection. **(A)** The pre-transplant chest CT is presented without obvious abnormality. **(B)** On 18 days post-transplant, abdominal CT examination revealed a mixed density mass shadow in the medial inferior right side of kidney graft, hematoma considered. **(C)** The chest CT revealed patchy shadows in the left lung on 38 days after transplantation. **(D)** After 7 days of initial treatment, the cavity formed in the left lung can be seen in radiological examination. **(E)** A chest CT showed consolidation of the left upper lobe with a ground-glass density shadow, multiple nodules in both lungs on 52 days. **(F)** A chest CT showed the cavity in the upper left lung had become larger than that in the previous examination 2 weeks earlier.

Hemodialysis was carried out intermittently due to post-operative continuous DGF. Eight days after surgery, a routine B-ultrasound showed a small amount of effusion on the upper lateral side of the graft. Ten days later, the patient reported peri-renal pain. An abdominal computed tomography (CT) examination revealed mixed density mass shadow in the upper and medial inferior right side of the kidney graft, hematoma was considered (Figure 2). The hematoma enlarged to 64 × 54 mm in the superior side and 95 × 37mm in the medial side in the next 2 days and had no significant changes shortly thereafter. Considering there was no indication for emergency operation, correct coagulation function, anti-infection, immunosuppression, and other conservative treatments were continued.

On 38 days post-transplantation, the chest CT revealed multiple small nodules and patchy shadows in the left and right lower lobes of the lung. In addition, the patient was positive for serum fungal galactomannan (GM) detection (1.176 μg/L, the normal reference value is <0.5 μg/L) and had an elevator procalcitonin at 0.442 ng/ml (<0.05 ng/ml is normal value) with normal body temperature and no other obvious clinical symptoms. The patient was regarded as having a pulmonary infection and treated with caspofungin (50 mg, qd), voriconazole (200 mg, q12 h), sulperazon (1.5 g, q12 h), and ganciclovir (250 mg, qod) by intravenous drip. A bronchoalveolar lavage was performed subsequently, which showed pulmonary mucosa was slightly congested without obvious secretion. Oral anti-immune rejection medications were reduced to tacrolimus 0.5 mg/12 h, mycophenolate mofetil 0.25 g/12 h, and methylprednisolone 24 mg/d.

**Figure 3:**
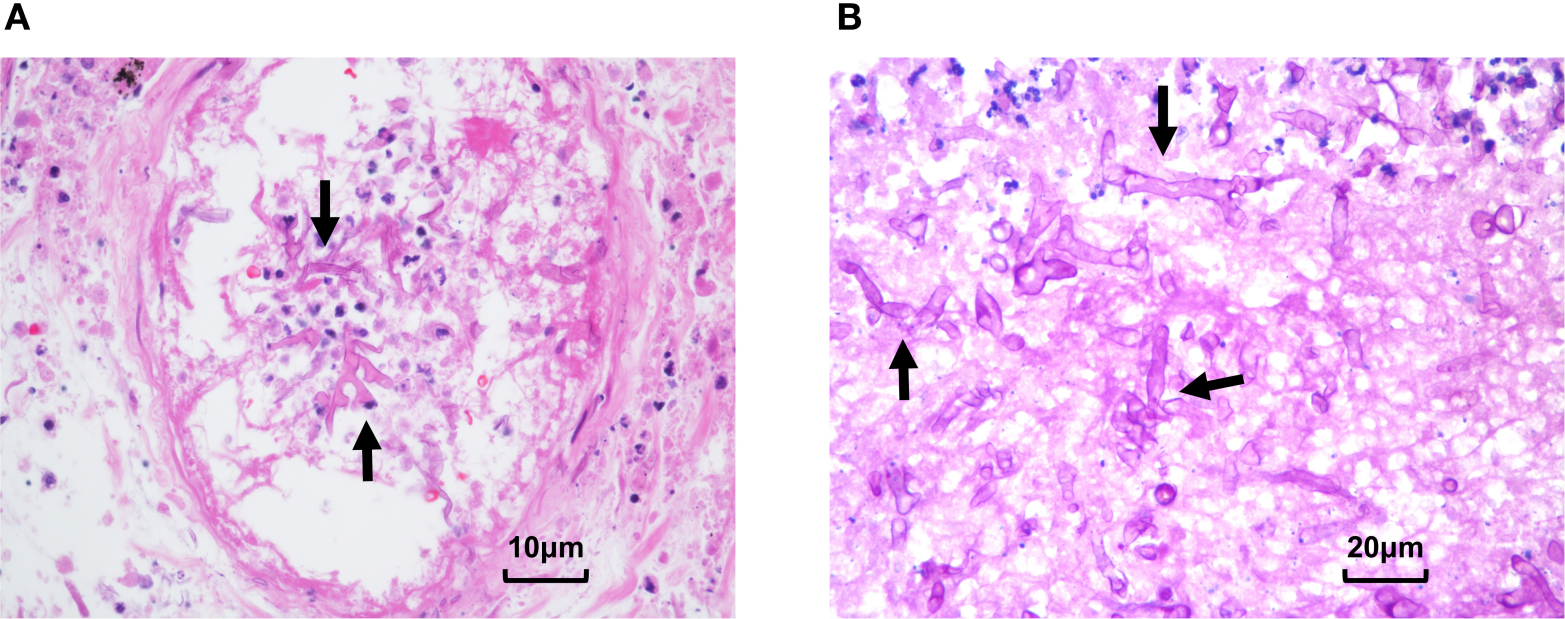
Pathological presentation of pulmonary and splenic mucormycosis. **(A)** Percutaneous lung biopsy showed alveolar epithelial hyperplasia, interstitial hyperemia, inflammatory cell infiltration, and fungal hyphae and spores can be seen in necrotic tissues. **(B)** Pathological results of spleen tissue showed multifocal necrosis was formed under the spleen capsule with mycelia and spores (Arrow: mycelia).

After seven days of treatment, radiological examination revealed new lesions and the cavity formation in the upper left lung. Based on the examination, multidisciplinary consultation advised that fungal lung infections were possible (aspergillus or mucor), which needs early and definitive diagnosis by biopsy. However, after being informed of the possible complications of lung biopsy, the patient and family refused to biopsy and requested conservative treatment in consideration of the current coagulation profile. The voriconazole was changed to amphotericin B liposome, and cotrimoxazole was treated to prevent bacterial infections. After continuous treatment for several days, the patient began to develop hemoptysis and dark red secretions in the sputum. A chest CT showed consolidation of the left upper lobe with ground-glass opacity and multiple nodules in both lungs. Following informed consent, a CT-guided percutaneous lung biopsy was immediately performed to identify the pathogen under regional anesthesia. The results indicated that fungal hyphae and spores could be seen in the biopsies with a high suspicion of mucormycosis (Figure 3A). All oral immunosuppressants were stopped except methylprednisolone, which was gradually reduced to 4 mg/d. Following multidisciplinary consultation and discussion with the patient and relatives, he agreed to abandon the graft immediately and underwent embolization of the transplanted renal artery instead of a transplant nephrectomy to avoid the potential risk of intolerance of surgery. The antibody combination was adjusted as following: amphotericin B liposome (50 mg, qd), posaconazole (400 mg, q12 h), and meropenem (1 g, q8 h). Ten days later, a chest CT showed the cavity in the upper left lung as larger than that in the previous examination. On 78 days after transplantation, the patient suffered acute abdominalgia accompanied by vomiting, high fever, shock, and confusion, meanwhile, the blood pressure dropped to 64/45 mmHg. An emergency exploratory laparotomy was performed and showed a hemorrhage of the spleen, which was then cut off. The pathological results of spleen tissue submitted revealed multiple necroses under capsule with visible mycelium and spores of mold (Figure 3B). Despite initial remarkable improvements, the patient developed a round-like mixed slightly low-density shadow in his right cerebellum showed in a brain CT scan on 85 days post-transplant. The patient and his family abandoned any treatment and soon left the hospital. His death was confirmed a few days after follow-up.

## Discussion

In recent years, the incidence of pulmonary fungal infections in the clinic has increased rapidly, especially in kidney transplantation recipients (11). The risk factors of pulmonary mycosis post-transplant include further surgery, administration of immunosuppressants, and the short-time use of or without anti-inflammatory drugs (12, 13). To date, fungal infection was still the leading cause of early death in solid organ transplantation recipients (14, 15). Mucor is a rare conditioned pathogen that can severely endanger hypo-immune recipients using various immunosuppressants. Here, we report the complete episode, diagnosis, and treatment process of pulmonary mucormycosis in the patient who underwent a kidney transplantation.

In this patient, DGF and clinical potential graft rejection may be reasons for hematomas appearing early after surgery and compression of the transplanted kidney by a peri-renal hematoma may also exacerbate graft ischemia, delay functional recovery, and limit the patient's absolute bed rest, additionally leading to increased water load and the risk of pulmonary infection (16). It is therefore necessary to discuss the treatment of peri-renal hematoma associated with post-operative transplantation. Peri-renal hematoma is an early complication after renal transplantation, associated with coagulation dysfunction caused by chronic uremia, vascular anastomosis, blocked drainage, and early activity in post-operative patients (12, 17), which may compress blood vessels and renal parenchyma as well as cause oliguria, promote DGF, and even lead to graft loss (18–20). Timely treatment of post-operative peri-renal hematoma is essential. However, there exists a controversy regarding treatment, conservative treatment such as anti-rejection and hemodialysis or surgical intervention like percutaneous drainage, surgical decortication, or laparoscopic intervention are all applicable without definite indications (21, 22). In short, we all agree that expanding hematoma, affecting the blood flow and function of graft, should be explored and evacuated opportunely (23).

Mucormycosis develops rapidly, has a poor prognosis, and is rarely reported in kidney recipients (24). Considering that mucoromycetes are pathogens that are present in the environment and cause opportunistic infections in immunocompromised individuals, we first identified the foci of infection in the lungs and confirmed the presence of mucor in lung tissue biopsies, the source of this mucormycosis was likely to be the pulmonary infection. In this case, it takes just 15 days from patchy shadows seen in radiological examination to large cavity formation and splenic rupture with fungal infections 25 days later. Early and reliable diagnosis along with the identification of pathogens is necessary for treatment (25). This patient with idiopathic peri-transplantation hematoma, hard on palpation, and abnormal coagulation, a kidney biopsy may result in aggravation of peri-transplantation hematoma or even a rupture of the transplanted kidney. We used lung biopsy and spleen histopathology to confirm the diagnosis of mucormycosis, which usually manifested as negative in the serological examination and non-specific clinical presentations (26). Many researchers are committed to finding new methods for making the diagnosis of mucormycosis earlier leading to improve survival. A previous study reported that mucorales specific T cells detected by an enzyme-linked immunospot can be a surrogated diagnostic marker (27). Molecular assays such as conventional polymerase chain reaction, DNA sequence, and melt curve analysis provide an alternative auxiliary method for the diagnosis (28–30). More attention and effort should be paid to the early diagnosis of mucor infection. It is currently agreed that only amphotericin B and its liposomes, and posaconazole are effective medicines for treating mucor (31). The liposomal amphotericin B is recommended as a first-line therapy because of less renal toxicity (32, 33). A retrospective cohort study of antifungals among inpatients of invasive aspergillosis or mucormycosis highlighted the importance of invasive fungal infection monitoring during hospitalization and the use of appropriate prophylaxis and treatment (34). Other cases have also pointed out that pre-emptive treatment is critical for the rarely seen mucormycosis in organ transplantation recipients (35, 36). There is a contradiction between the treatment of mucormycosis and the use of immunosuppressants against graft rejection. Firstly, immunosuppression promotes the invasion of pathogens. On the other hand, drug-drug interactions, the dose of tacrolimus should be reduced by 60–75% when receiving posaconazole typically (37), which should be evaluated carefully (31). At the early stage of infection, the effective dose of immunosuppressants can be maintained. With the development of the disease, cell proliferation inhibitors should be stopped in time. Then, all inhibitors other than glucocorticoids should be stopped immediately with the rapidly developing and refractory infections. Considering the more than 60% mortality rate in pulmonary mucormycosis after kidney transplantation (38–40), abandoning the graft is a way to reduce loss and advance survival.

In conclusion, we reported the case of pulmonary mucormycosis in kidney transplantation recipients. Prevention and early diagnosis are essential due to the uncontrollable progression of mucor infection. The authors reviewed three key treatments including rational antifungal therapy, timely elimination of immunosuppressants, and abandoning the graft if necessary.

## Ethics Statement

Written informed consent was obtained from the individual(s) for the publication of any potentially identifiable images or data included in this article.

## Author Contributions

HZ and KW reviewed the case and wrote the article. HC collected the clinical data and revised the report. LS and ZW were involved in collecting clinical data and providing related literature. SF participated in the post-operative management of this case. RT and MG led the treatment of the patient and revised the manuscript. All authors contributed to the article and approved the submitted version.

## Conflict of Interest

The authors declare that the research was conducted in the absence of any commercial or financial relationships that could be construed as a potential conflict of interest.
